# Well‐controlled mucosal exudation of plasma proteins in airways with intact and regenerating epithelium

**DOI:** 10.14814/phy2.16096

**Published:** 2024-06-04

**Authors:** Carl Persson

**Affiliations:** ^1^ Laboratory Medicine University Hospital of Lund Lund Sweden

**Keywords:** epithelial regeneration, first line defense, human airway mucosa, maintained epithelial barrier, microvascular‐epithelial cooperation, plasma exudation

## Abstract

Superficial, systemic microcirculations, distinct from the pulmonary circulation, supply the mucosae of human nasal and conducting airways. Non‐injurious, inflammatory challenges of the airway mucosa cause extravasation without overt mucosal oedema. Instead, likely reflecting minimal increases in basolateral hydrostatic pressure, circulating proteins/peptides of all sizes are transmitted paracellularly across the juxtaposed epithelial barrier. Thus, small volumes of extravasated, unfiltered bulk plasma appear on the mucosal surface at nasal and bronchial sites of challenge. Importantly, the plasma‐exuding mucosa maintains barrier integrity against penetrability of inhaled molecules. Thus, one‐way epithelial penetrability, strict localization, and well‐controlled magnitude and duration are basic characteristics of the plasma exudation response in human intact airways. In vivo experiments in human‐like airways demonstrate that local plasma exudation is also induced by non‐sanguineous removal of epithelium over an intact basement membrane. This humoral response results in a protective, repair‐promoting barrier kept together by a fibrin‐fibronectin net. Plasma exudation stops once the provisional barrier is substituted by a new cellular cover consisting of speedily migrating repair cells, which may emanate from all types of epithelial cells bordering the denuded patch. Exuded plasma on the surface of human airways reflects physiological microvascular‐epithelial cooperation in first line mucosal defense at sites of intact and regenerating epithelium.

## 1 INTRODUCTION

**FIGURE 1:**
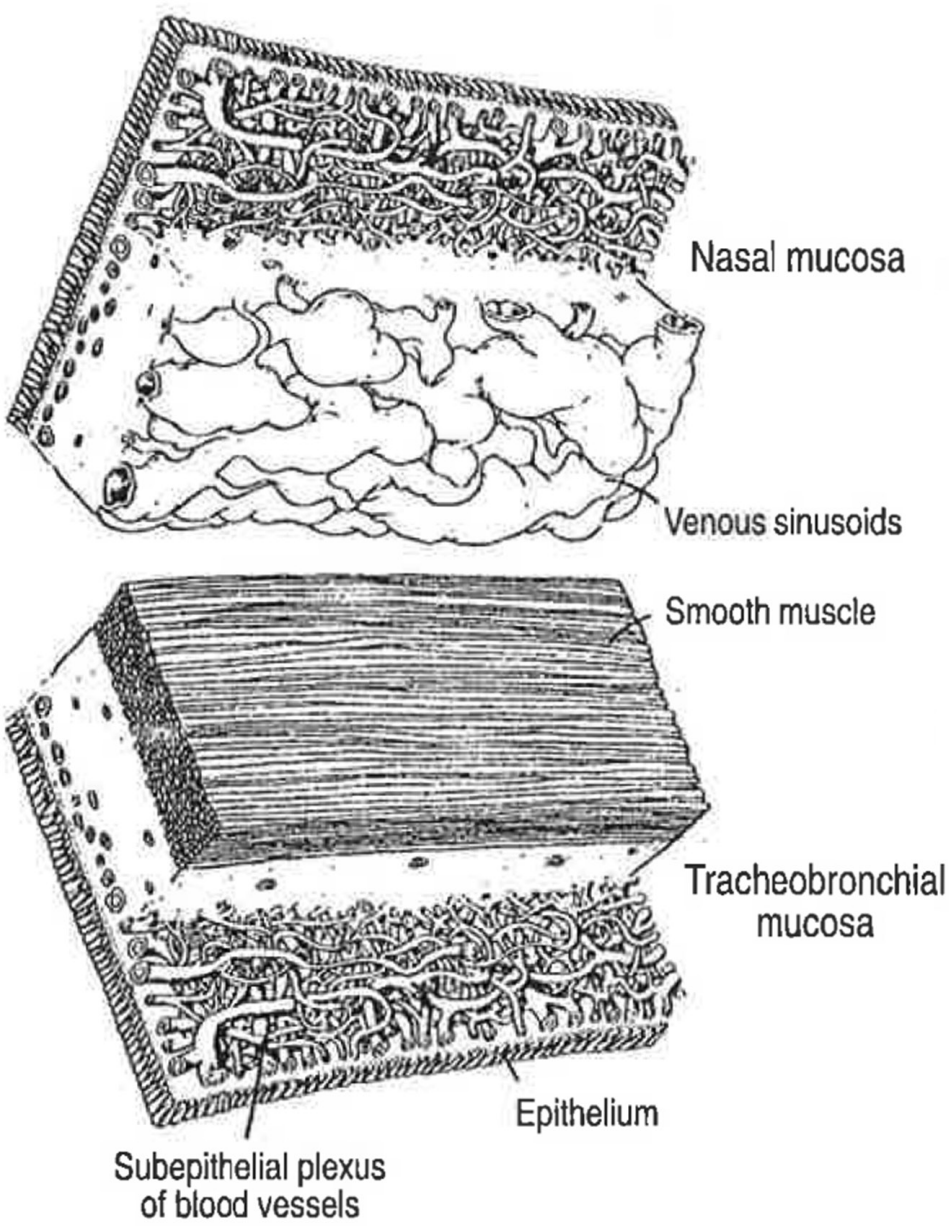
Throughout the human nasal and tracheobronchial airways, profuse, highly responsive, systemic microcirculations are juxtaposed with pseudostratified epithelial linings. As illustrated, the “Epithelium” and the “Subepithelial plexus of blood vessels” of the conducting airways (“Tracheobronchial mucosa”) are similarly organized in the “Nasal mucosa.” However, filling of “Venous sinusoids” and constriction of circular “Smooth muscle,” respectively, regulate size of the free lumen in human nasal and tracheobronchial airways. (Previously published, Persson, Svensson, et al., 1992). The guinea‐pig trachea is equipped with a similar subepithelial microcirculation positioned just beneath a pseudostratified epithelial lining (Erjefält et al. 1994, 1996).

The human lung has two blood circulations. A low‐pressure pulmonary circulation receives the cardiac output from the right heart. It is involved in gas exchange at the alveolar level and delivers oxygenized blood to the left heart. Additionally, the lung and bronchi are supplied by the systemic bronchial circulation. Arising from the aorta it delivers about 1% of the cardiac output (Deffebach et al., 1987). The mucosa of human nasal, tracheal, and bronchial airways (Figure 1), like that of guinea‐pig and rat trachea (Erjefalt et al., 1996; McDonald, 1994), is similarly supplied by superficial, systemic microcirculations. The mucosal microcirculations carry more than oxygen, cells, and fluid. Protein‐peptide systems with established roles in defense and repair (cathelicidins, complement proteins, fibrinogen, fibronectin, mannose‐binding lectin, natural antibodies, and pentraxins) also circulate close to the basal aspect of the epithelium in human nasal and tracheobronchial airways and will be released at mucosal provocations.

The physiology and structural correlates of extravasation of plasma induced by challenge of systemic microcirculations with histamine‐type autacoids has been established (McDonald, 1994; Svensjo et al., 1979): Vaso‐permeability autacoids induce dose‐dependent, reversible, and reproducible gap formation between venular endothelial cells. Through these gaps, a portion of all‐size circulating molecules with associated liquid (not cells) leave the circulation. To date there has been little discussion in the literature about the passage of plasma proteins across the epithelium in nasal and bronchial airways, which is the focus of this article.

Movement of plasma proteins to the mucosal surface of human challenged airways is mostly reported merely as evidence of increased vascular permeability (Pizzichini et al., 1997; Stockley et al., 1979). Lack of attention to the crucial epithelial passage may reflect the belief that appearance of the circulating proteins on the surface of challenged or diseased airways is a second line of response operating in airways with mucosal injury when the epithelial barrier is lost (Korsrud & Brandtzaeg, 1983). There are further reasons for the neglect. For example, airway exudation of plasma is absent in frontier mucosal research involving cell cultures, clinical cell biology, and mouse models (lacking a bronchial circulation, Eldridge & Wagner, 2019). In accord, airways plasma exudation, as a component of mucosal immunity and epithelial regeneration, respectively, is rarely considered in literature covering these fields (Box 1). On this note, it is not known how current notions of molecular regulation of epithelial permeability (e.g., Sidhaye et al., 2008) and epithelial regeneration (e.g., Jin et al., 2022; Kicic et al., 2010), respectively, will fare when validated in vivo in plasma exudation‐milieus.

BOX 1 **Traditional perspectives/views on plasma exudation responses:**

- Plasma exudation is viewed as mucosal pathology with increased microvascular permeability and loss of the protective epithelial barrier
- Plasma exudation is not discussed in leading literature on respiratory innate immune defense
- Plasma exudation is absent in fundamental research approaches in the fields of interest (immune defense and epithelial regeneration) involving cell culture, clinical cell biology, and mouse models
- A major antimicrobial peptide like cathelicidin is believed to be produced exclusively by local cells at airway challenges

**The human airways in vivo perspective on plasma exudation responses:**

- Physiological microvascular‐epithelial cooperation leads to non‐injurious epithelial transmission of all‐size plasma peptides/proteins without reducing the protective epithelial barrier that prevents penetration of inhaled molecules
- Several major antimicrobial and repair‐promoting peptides/proteins, including cathelicidin, currently believed to be produced by local cells, actually emerge as components of plasma exudates
- The in vivo physiology of airways plasma exudation qualifies this response for a first line respiratory antimicrobial mechanism in airways with intact epithelial lining
- A local plasma‐derived provisional barrier and molecular milieu protect patchy sites of epithelial denudation in human‐like airways and promote speedy epithelial regeneration until a cellular barrier is restituted

This review is concerned with the bigger picture of microvascular‐epithelial interaction in vivo in human and human‐like airways. As summarized, different approaches are employed to obtain insight into in vivo physiology of mucosal exudation of circulating proteins in human airways, including the relatively inaccessible bronchi. The focus is on appearance of the plasma proteins on the surface of the intact airway mucosa in vivo. Next, experimental studies unveiling the modus operandi of interaction between extravasated plasma and the epithelial barrier in intact airways are presented. The picture is complemented with in vivo events, including plasma exudation, following common types of epithelial shedding that leave a patch of naked but uninjured basement membrane. A novel physiological framework emerges for interpreting occurrence plasma peptides/proteins in surface liquids of human challenged airways (Box 1).

## 2 APPROACHES TO ELUCIDATE MOVEMENT OF PLASMA PROTEINS TO THE SURFACE OF CONDUCTING AIRWAYS IN HUMANS

### 2.1 Strategy

In search for a translational in vivo model to study the fate of proteins extravasated from airway mucosal microvessels the guinea‐pig trachea has increasingly been recognized as an appropriate species. Like human bronchi, the guinea‐pig trachea harbors a pseudostratified epithelial lining juxtaposed by a superficial, systemic microcirculation. Even more importantly, the fate of proteins extravasated in response to mucosal challenges appears to be controlled by similar physiologic mechanisms in the guinea‐pig trachea as in human airways in vivo (vide infra).

To understand events in the less accessible human bronchi in vivo, the following approaches have been developed: Early exploratory findings in guinea‐pig trachea were validated, complemented, and expanded by studies in the human nose. Both approaches involved controlled conditions scarcely achievable in the human bronchi in vivo. However, guinea‐pig tracheal mechanisms and human nasal observations (Persson et al., 1992) may represent well the mucosal exudative response of human bronchi.

### 2.2 Physiological baselines for in vivo studies of intact airways mucosa

Optimal, physiological baselines for in vivo studies of airway mucosal responses require no physical contact with the mucosa except fluids containing test agents and biomarkers. Initial washing liquids, applied to produce baseline starting conditions prior to administration of challenge solutions, increase the possibility of detecting exudative responses. For example, significant dose‐dependent protein extravasation effects of locally applied leukotrienes were demonstrated already at 1 and 5 pmol (Persson et al., 1986) compared to the reported need of 0.1–100 nmol doses in original studies of this effect in guinea‐pig trachea (Woodward et al., 1983).

Concentrations of challenge agents and absorption tracers in contact with the mucosa, the exposed mucosal surface area, and duration of challenge and absorption, respectively, were controlled. Further, the same area was exposed to challenge agents and absorption tracers as well as subsequently lavaged for sampling surface molecules and stopping absorption, respectively. Desired approaches were achieved in guinea‐pig trachea (Erjefalt et al., 1985; Erjefalt & Persson, 1989) and even more so in the human nose (Greiff et al., 1990). Mucosal application of pieces of filter paper or other kinds of absorbing discs have some use in studies of airway mucosal challenge and sampling. It is a simple and popular mode purported to sample “mucosal surface indices.” However, reflecting the large size of the filter paper compared to the normal height of airway surface liquids, fluid will likely be sucked across the epithelium. It is of note that a filter paper is about 10 times as thick as the airway surface liquid is deep. Distinct from the lavage method, the absorbing discs will distort baseline mucosal barrier properties and sample also subepithelial solutes including macromolecules (Erjefält & Persson, 1990).

### 2.3 Studies of effects in vivo induced solely by loss of epithelial cells from an intact basement membrane

Environmental challenges may cause shedding of bronchial epithelial cells without bleeding and without injury to the basement membrane like it occurs in asthma. The response to such epithelial loss alone needed investigation in vivo in an accessible, human bronchi‐like mucosa (Erjefalt et al., 1995a; Persson & Erjefält, 1997). By careful mechanical removal of tiny stretches of epithelium in non‐inflamed guinea‐pig trachea, sharply defined areas of denuded basement membranes were produced (Erjefalt et al., 1995a). Importantly, no bleeding occurred and the basement membrane remained intact. Inflammatory challenge‐induced tiny craters of epithelial loss were also studied (Erjefält et al., 1997a). Questions regarding mucosal physiology and pathophysiology were asked in these in vivo experiments. Human bronchi in vivo were not accessible for similar experimental studies, but guinea‐pig in vivo observations on epithelial loss‐regeneration were checked in part against known features of desquamative bronchial disease.

### 2.4 Human bronchial challenge and sampling bronchial surface indices

In human bronchi in vivo, inhalation or locally applied challenges using bronchoscopy were followed by sampling of induced sputum and bronchial lavage fluid, respectively (Halldorsdottir et al., 1997; Salomonsson et al., 1992). Considering risks for undue inflammatory effects (Huang et al., 2006), repeated bronchial lavages could not be used to produce baseline starting conditions in human bronchial in vivo studies. Of note, this limitation means that albumin levels in bronchial surface liquids may not reliably reflect plasma protein exudation responses (vide infra).

## 3 UNEXPECTED RESULTS OBTAINED WITH THE GUINEA‐PIG TRACHEA, A MODEL OF HUMAN AIRWAYS, SUBJECTED TO MUCOSAL CHALLENGES IN VIVO

Widely adopted traditional views maintain that airway plasma protein exudation reflects oedema, injury, and general penetrability of the epithelial lining. Hence, a string of surprising results was obtained in vivo in guinea‐pig trachea that harbors a systemic mucosal microcirculation with microvessels juxtaposed with a pseudostratified epithelium similar to human nasal and bronchial airways (Figure 1).

### 3.1 Overt mucosal oedema was not detected at challenge‐induced mucosal exudation of plasma

Topical mucosal challenges with histamine‐like autacoids and inflammatory toxins/allergens produced venular inter‐endothelial gaps just beneath the epithelium causing release (extravasation) of non‐sieved plasma proteins. Following the outpouring of circulating solutes, the lamina propria became endowed with plasma proteins. However, it was not associated with increased mucosal wet to dry weight ratio, mucosal thickening, or distended interepithelial spaces (Erjefalt et al., 1995b; Persson et al., 1986). Hence, no overt oedema occurred. Nor was elimination of extravasated proteins by lymphatics detected (Erjefalt et al., 1993). It emerged that extravasated macromolecules moved all‐around epithelial cells towards the mucosal surface in the area of interest (Erjefalt et al., 1995b). It is conceivable that bulk plasma exudate utilized the entire interepithelial circumferences in the area of interest for its transepithelial passage. However, the crucial path through the apical tight junction belt, with all its strands and grooves, remains to be mapped and understood.

### 3.2 Distinction between guinea‐pig and mouse tracheal responses

Challenge of guinea‐pig tracheal mucosa with histamine‐like autacoids produced prompt, concentration‐dependent microvascular‐epithelial exudation of plasma proteins. Flooding of the airways was not seen (Erjefalt & Persson, 1989). All employed, vasoactive, mucosal challenges in guinea‐pig trachea led to extravasated plasma macromolecules appearing in mucosal surface liquids (Erjefalt et al., 1995b; Erjefalt & Persson, 1989; Persson et al., 1998). By contrast, using a similar experimental approach and administering the same type of mucosal challenges, epithelial passage of extravasated plasma proteins was not detected in mouse trachea (Erjefält et al., 1998).

### 3.3 Plasma proteins move across a morphologically intact pseudostratified epithelium

As observed by light microscopy, transmission electron microscopy, and scanning electron microscopy, the human‐like pseudostratified epithelial lining of guinea‐pig trachea was not appreciably altered by challenge with histamine‐like autacoids producing microvascular‐epithelial exudation of plasma macromolecules (Erjefalt et al., 1995b). Hence, histological observations suggested that airways plasma protein exudation is a well‐regulated physiological response. In support, Serikov et al. (2002), employing scanning electron microscopy of rat tracheal mucosa, then reported lack of ultrastuctural changes of the epithelium in experiments involving vagal stimulation‐induced exudation of albumin‐sized plasma tracers.

However, further questions needed answers: what size‐selectivity applies to challenge‐induced protein exudation and, perhaps most importantly, what happens to epithelial penetrability in the opposite, inward direction?

### 3.4 Lack of size restriction at microvascular‐epithelial exudation of proteins

**FIGURE 2:**
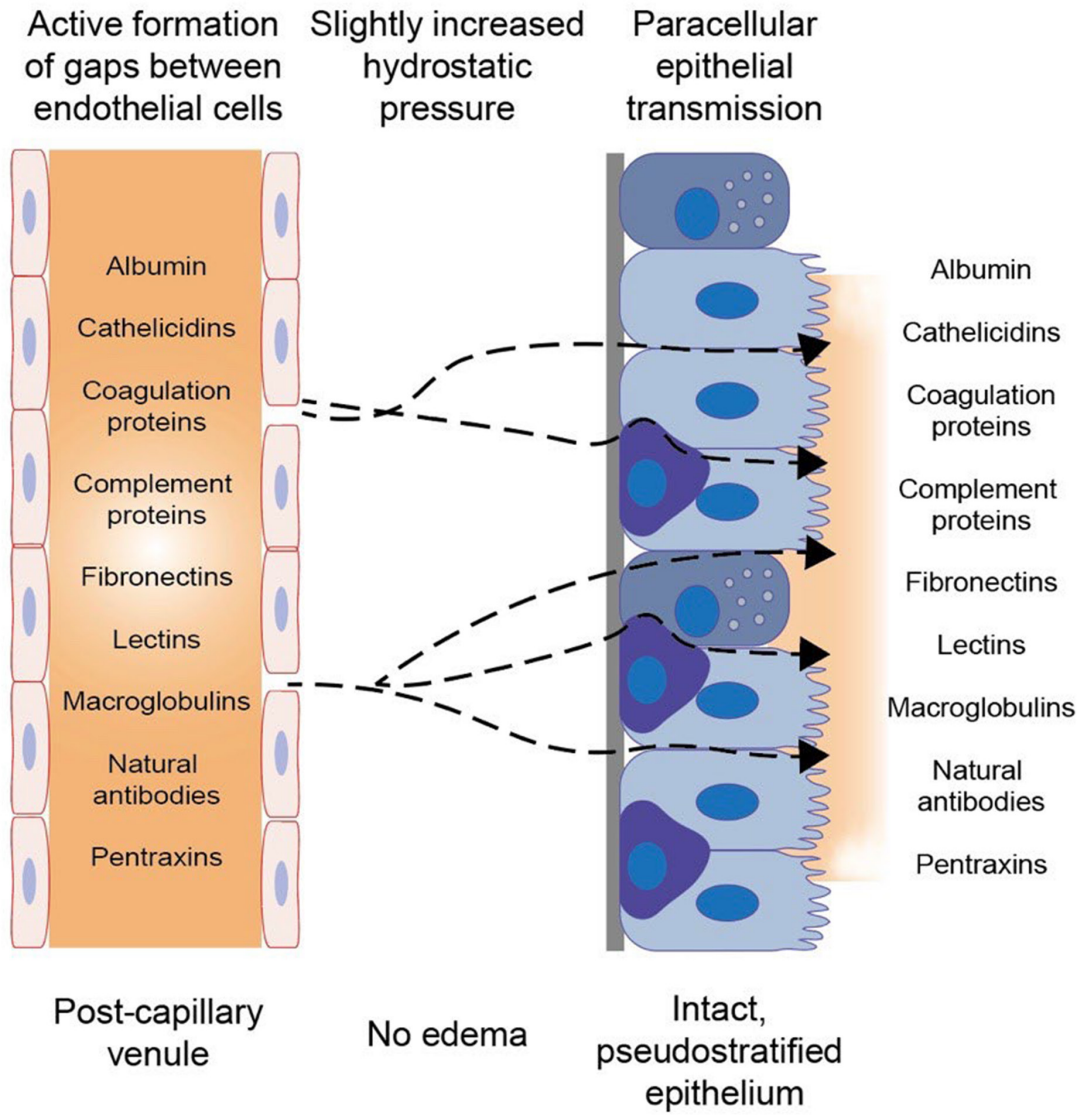
Schematic illustration of the pathway for microvascular‐epithelial exudation of bulk plasma (broken arrow lines) that agrees with observations in vivo in guinea‐pig trachea and human airways. The intact pseudostratified epithelium is represented by six ciliated cells, two secretory cells, and three basal cells. Mucosal challenges by toxins and infections cause extravasation of bulk plasma through actively regulated inter‐endothelial gaps. The extravasated bulk plasma enters the lamina propria and apparently needs to increase the epithelial basolateral hydrostatic pressure by only 5 cm H_2_O or less to drive an unfiltered proteinaceous exudate across the pseudostratified epithelial lining through paracellular pathways. The epithelial passage occurs without overt distention of lateral intercellular spaces and without injury. In vivo, plasma‐derived macromolecular tracers move all‐around epithelial cells up to tight junctions in the area of interest. However, the crucial pathways through the tight junctional belt with all its grooves and ridges remain to be defined and understood to comply with the passage of bulk plasma exudates without altering the mucosal barrier function against penetration of inhaled molecules. Since plasma proteins/peptides are exudated without size restriction, the listed wide range of circulating antimicrobial molecules will also appear on the mucosal surface at the site of challenge. This figure is a slight modification of a previously published version (Persson, 2019b).

Size restriction applies to human bronchial lumen entry of plasma proteins in health but is lost at chest infections with equal exudation of albumin and α_2_‐macroglobulin (Stockley et al., 1979). In guinea‐pigs autacoid mucosal challenges induced exudation of circulating ^125^iodine‐fibrinogen (>350 kDa) along with fluorescein‐labeled dextran (an uncharged macromolecule, 150 kDa) and ^131^iodine‐albumin (70 kDa). The three distinctly sized exudation tracers exhibited about the same concentration ratios in the mucosal surface exudate as in circulating blood (Erjefalt & Persson, 1989). The data demonstrated lack of molecular sieving during the transmission of macromolecules across endothelial and epithelial barriers including their basement membranes. Hence, mucosal challenge with simple, non‐injurious autacoids caused physiologic, well‐controlled microvascular‐epithelial transmission of circulating macromolecules without signs of size restriction (Figure 2). As reflected by some passage of the smaller plasma tracers but not ^125^iodine‐fibrinogen into the mucosal lavage liquids, size restriction applied at baseline (Erjefalt & Persson, 1989). A limited outward leakage of albumin at baseline also occurs in human bronchi in vivo (Stockley et al., 1979). Airways are considered to have a “leaky epithelium” but this is based on passage of electrolytes (Saint‐Criq & Gray, 2017) rather than protein‐sized molecules. Of experimental importance, technetium 99 m labeled diethylenetriamine pentaacetate (DTPA) (492 Da) is similarly absorbed at a low rate at baseline in both human and guinea‐pig airways (Greiff, Erjefält, et al., 1991).

### 3.5 Pivotal barrier asymmetry: Outward vs inward penetrability of the protein‐exuding airway epithelium

Crucially, the intact structure of protein‐exuding epithelium (Erjefalt et al., 1995b) was associated with an intact epithelial barrier function as regards inward penetrability of molecules deposited on the challenged mucosal surface (Erjefält & Persson, 1991a). Thus, absorption of ^131^iodine‐albumin across the tracheal mucosa was not significantly increased by topical exudative challenges. Mucosal challenges with bradykinin and, in sensitized animals, allergen produced mean exudation of plasma 15 and 24 times, respectively, above control (p < 0.001). By contrast, the mean absorption data under identical conditions were 1–1.5 times (p > 0.05) the control conditions in airways subjected to these two challenges. Furthermore, repeat absorption measurements, starting as soon as the plasma exudation response to bradykinin had stopped and continuing for an additional 30 min, demonstrated lack of increased absorption penetrability in the immediate post exudation phase (Erjefält & Persson, 1991a). Additionally, Greiff, Erjefält, et al. (1991) demonstrated that histamine‐induced plasma protein exudation changed neither the absorption of ^131^‐iodine‐albumin nor technetium 99 m labeled DTPA (492 Da). Such epithelial barrier asymmetry would be decisive for a well‐functioning role of plasma protein exudation in first line mucosal defense (Figure 2).

## 4 CONFIRMATION IN HUMAN AIRWAYS OF PLASMA PROTEIN EXUDATION IN HEALTH AND DISEASE

Major features of mucosal challenge‐induced microvascular‐epithelial transmission of plasma proteins in guinea‐pig trachea were confirmed in human airways in vivo, first in nasal airways and then in the bronchi.

### 4.1 Prompt, dose‐dependent, fully reversible, and repeatable response

Exudation of plasma proteins in response to topical histamine challenges of human nasal mucosa was prompt, concentration‐dependent (Greiff et al., 1990), reversible, and repeatable (Svensson et al., 1989). These observations support the physiologic nature of the epithelial transmission of plasma proteins that follows upon their extravasation.

### 4.2 No size restriction

Studying human allergen‐challenged nose, Baumgarten et al. (1985) had demonstrated lack of size restriction of plasma proteins up to the size of high molecular weight kininogen (110 kDa, but when circulating in complex with prekallikrein the molecular weight would amount to 280 kDa). The guinea‐pig data discussed above significantly expanded the range of protein sizes that were exuded without sieving. Confirming and further expanding the guinea‐pig data, Greiff et al. (2002) demonstrated non‐sieved exudation of albumin together with the 700 kDa protein α_2_‐macroglobulin in human nasal airways subjected to non‐injurious histamine challenges. In accord, exudation of α_2_‐macroglobulin and fibrinogen occurred promptly at bronchial inflammatory challenges (Berman et al., 1996; Halldorsdottir et al., 1997; Salomonsson et al., 1992).

### 4.3 Direction‐dependent asymmetry of airways mucosal barrier function

Simple comparisons between exudation and absorption of tracer molecules are used to indicate mucosal barrier asymmetry. In human nasal studies it was demonstrated that even a high exudative concentration of histamine (2 mg/mL; Greiff et al., 1990) was not associated with any change in mucosal absorption of molecules deposited on its surface (Greiff, Wollmer, et al., 1991). The nasal absorption of ^51^Cr EDTA over 15 min, determined by urinary recovery of radioactivity, corresponded to 0.095 (SE 0.023) ml of the instillate without histamine. Histamine did not change this absorption index (0.093 mL SE 0.025). Additional experiments were carried out confirming specificity of the data as regards absorption across the nasal mucosa (Greiff, Wollmer, et al., 1991).

The epithelial barrier asymmetry is expressed in human inflammatory diseases. Lack of increased penetrability, rather a significantly decreased absorption (sic!) of different tracers (^51^Cr EDTA and desmopressin) occurs in seasonal allergic rhinitis exhibiting sustained exudation of large plasma proteins including α_2_‐macroglobulin (Greiff et al., 1993, 1997). The reduced absorption penetrability is consistent with historical observations in allergy (see Greiff et al., 1993), but has not been well explained. Human bronchial asthma is characterized by increased bronchial mucosal surface levels of plasma proteins such as α_2_‐macroglobulin without signs of increased absorption of inhaled molecules (reviewed in Persson, 2019b; Persson, 2021). The most frequently used bronchial absorption tracer in asthma has been technetium 99 m labeled DTPA (492 Da), which also was used to demonstrate plasma exudation without increased absorption in guinea‐pig trachea (Greiff, Erjefält, et al., 1991). Thus, the pseudostratified epithelium of human nasal and bronchial airways, like that of the guinea‐pig trachea, lets through plasma macromolecules whilst maintaining its normal barrier function against inhaled molecules (Figure 2). Importantly, as suggested by observations in nasal airways infected by coronavirus 229E (Greiff et al., 1994) the barrier asymmetry extends to airways infection.

### 4.4 Albumin in human bronchial surface liquids is not always an ideal plasma exudation tracer

Under baseline conditions in human bronchi (Stockley et al., 1979) and guinea‐pig trachea (Erjefalt & Persson, 1989), molecules of the size of albumin but not large plasma proteins, such as fibrinogen and α_2_‐macroglobulin, seep across mucosal endothelial and epithelial barriers. Thus, depending on how well mucosal surface clearance mechanisms operate, albumin will variably accumulate in airway surface liquids. By rinsing the mucosal surface prior to challenge, creating negligible baseline levels of albumin, this specificity problem can be solved in the human nose and guinea‐pig trachea (vide supra), but not in human bronchi where repeat lavages may cause inflammation (Huang et al., 2006). Hence, bronchial albumin levels have not indicated a significantly increased plasma protein exudation in human bronchi challenged with allergen (Salomonsson et al., 1992) and bradykinin (Berman et al., 1996) where marked exudation of large plasma proteins such as fibrinogen is demonstrated. Exudation of fibrinogen rather associates with exudation of α_2_‐macroglobulin (Persson, 2019b; Stockley et al., 1979). Yet, albumin remains a common marker of plasma exudation (Wilson et al., 2002), although not an ideal one.

### 4.5 Bronchial exudation of plasma proteins may not reflect mucosal oedema

In a well‐controlled study, bradykinin challenge‐induced bronchial exudation of plasma proteins was not associated with increased airflow resistance (Berman et al., 1996) as would have been expected if a mucosal oedema had been present and encroaching on the free bronchial lumen. Lack of oedema tallies with the guinea‐pig data discussed above. Further, bronchial mucosal oedema may not characterize asthma that exhibits both plasma exudation and a normal epithelial absorption barrier (Chu et al., 2001; Persson, 2019b).

### 4.6 Blood flow changes may not be critical for plasma exudation responses

Histamine challenge‐induced plasma exudation in different test systems, including guinea‐pig trachea and human bronchi in vivo, was reduced by topical/inhaled beta‐agonists known to reduce endothelial gap formation and simultaneously increase the local blood flow (Erjefält & Persson, 1991b; Greiff et al., 1998). This observation thus supports a role of physiologic regulation of venular endothelial gap formation in airway mucosal microcirculations. However, topical treatment with beta‐agonists has not reduced plasma exudation in complex inflammatory conditions of airways diseases (Ahlström Emanuelsson et al., 2007) suggesting that the endothelial beta‐receptor function is more of theoretical interest than contributing to the clinical actions of this class of drug. Furthermore, a nasal decongestant known to reduce mucosal blood flow by 50% has not reduced histamine challenge‐induced plasma exudation in the human nose (Svensson et al., 1992). Hence, moderate variations in the rich superficial blood flow of human airways mucosae seem uncritical for plasma exudation responses.

## 5 MECHANISM OF UNIDIRECTIONAL OUTWARD TRANSMISSION OF MACROMOLECULES ACROSS PSEUDOSTRATIFIED AIRWAY EPITHELIUM

Considering the concordance between guinea‐pig trachea and human airways regarding microvascular‐epithelial anatomy and mucosal responsiveness, it was of interest to use the guinea‐pig trachea to explore mechanisms involved in the extrusion of proteins.

### 5.1 The idea of epithelial protein passage being driven by the extravasated plasma itself

With all types of tracheal mucosal challenges nothing suggested that plasma proteins were extravasated without also crossing the epithelium. Similarly, interventions with drugs that reduced challenge‐induced exudation of proteins did not alter the coupling between extravasation and epithelial transmission. No challenge seemed to regulate the epithelial transmission once extravasation had occurred (Persson et al., 1991). If neither biologic action on the epithelial barrier function nor epithelial injury were involved, what other type of influence could be exerted?

The intravascular hydrostatic pressure drives extravasation of plasma through the opened venular gaps. To some extent the hydrostatic pressure would then increase at the basolateral aspect of the epithelial barrier. When approached from beneath, the structure of mucosal epithelial linings would readily allow fluid to move up between epithelial cells. At the time, interesting observations had been made in studies of isolated rabbit gallbladders. Increasing basolateral hydrostatic pressures caused distention of the intercellular epithelial spaces and paracellular transmission of electrolytes and fluid (Wright et al., 1972). Further work in rabbit gallbladder by van Os et al. (1979) demonstrated that epithelial hydraulic conductivity increased already at minimally increased subepithelial pressure. A pressure load of 5 mbar applied on the serosal aspect of rabbit gallbladders increased the serosal to mucosal movement of ^14^C‐ethylene glycol (MW 62 Da) almost two‐fold whilst the same serosal pressure decreased the mucosal to serosal movement of this molecule by 20% (van Os et al., 1979). In 1992, a study involving sheets of dog tracheal epithelium demonstrated that an outward flux of albumin‐rich fluid, induced by increased subepithelial hydrostatic pressure, was associated with “dilatation of lateral intercellular spaces, disruption of tight junctions, and submucosal oedema” (Kondo et al., 1992). This report aligned with and strengthened the traditional view of airways plasma exudation as a sign and cause of oedema, injury, and general perviousness of epithelial linings, far from any defense aspect.

### 5.2 Main questions addressed by a tracheal tube method

To preserve the structures supporting the airway epithelium, a method using the guinea‐pig tracheal tube with its rings of cartilage scaffolding was developed. A fluid‐filled tracheal tube was mounted to allow increased hydrostatic pressure to impact on the basal aspect of the epithelial lining. Special care was used to avoid edge injury when connecting the trachea with tubes used for altering hydrostatic pressure. Success was determined by negligible epithelial passage of tracer molecules into the tracheal lumen under baseline conditions (Persson et al., 1990).

The particular questions with regard to mechanisms involved in the present plasma exudation response were threefold. First, can a slight increase in basolateral hydrostatic pressure, which does not cause appreciable oedema nor alter the epithelial lining morphology, allow outward movement of macromolecular solutes? Second and perhaps most importantly, can hydrostatic pressure‐operated exudation of macromolecules and associated liquid proceed without altered absorption penetrability? Third, can it also leave an epithelial lining that fully has maintained its normal barrier function?

### 5.3 A hydrostatic pressure mechanism induces reversible and repeatable outward macromolecular passage across intact tracheal mucosa

Raising the subepithelial hydrostatic pressure by only 5 cm H_2_O caused epithelial passage of macromolecular tracers into the lumen of the intact guinea‐pig tracheal tube (Persson et al., 1990). The effect was prompt and reversible, lasting as long as the pressure was elevated. Furthermore, epithelial transmission was fully reproducible and several repeat pressure increases did not affect the morphology of the epithelial lining (Persson et al., 1990). This exudation effect closely mimicked the in vivo plasma protein exudation. Involvement of such a mechanism in the microvascular‐epithelial interaction tallies with a mandatory epithelial passage of extravasated plasma proteins. It further supports generalization of the physiological plasma exudation responses to different vascular permeability‐active mucosal challenges in human‐like airways with a pseudostratified epithelium.

### 5.4 Increasing subepithelial hydrostatic pressure induces outward epithelial macromolecular penetrability without altering inward penetrability of mucosal surface molecules

Of critical importance, it was demonstrated that epithelial penetrability of protein‐sized molecules (FITC‐Dextran MW 70000 Da) present on the epithelial surface was neither increased nor decreased by the application of a serosal to mucosal pressure gradient of 5 cm H_2_O in guinea‐pig tracheal tubes (Gustafsson & Persson, 1991). This finding strengthened in vivo relevance of the tracheal tube data. Furthermore, Serikov et al. (2002) demonstrated that an intraluminal hydrostatic pressure of 4.5 cm H_2_O suffices to prevent vagally induced albumin exudation in rat trachea in vivo. The neurogenic plasma exudation response does not translate to human airways (vide infra). However, as far as it represents a general inflammatory response, this observation further strengthens in vivo relevance of the guinea‐pig tracheal tube data, which were obtained with a pressure gradient from serosa to mucosa of 5 cm H2O (Gustafsson & Persson, 1991; Persson et al., 1990).

Based on findings in guinea‐pig trachea in vitro and in vivo, it was proposed that a plasma exudation‐induced hydrostatic pressure load transiently moves bulk plasma between epithelial cells. This mechanism would provide a direction‐selective and non‐injurious paracellular pathway for passage of macromolecules into the airway lumen without increasing penetrability of molecules in the opposite direction. Further, the possibility was raised that the extravasated plasma itself creates its one‐way outward penetrability without influence of autacoid‐ and drug actions on the epithelium (Gustafsson & Persson, 1991; Persson et al., 1990).

## 6 PATCHY SITES OF EPITHELIAL DENUDATION IN DISEASE AND AT CHALLENGES

Both exudative and desquamative features of bronchi in subjects with asthma make it immediately understandable why the notion of a defective epithelial barrier in this disease is deeply rooted (i.e., despite ample demonstrations of maintained epithelial barrier in vivo against penetrability of inhaled molecules; reviewed in Persson, 2019b, 2021). As discussed above, the exudative passage of large plasma proteins should no longer be an argument since it likely reflects a strict asymmetric barrier function of the intact pseudostratified epithelium. However, equally tantalizing is the combination of maintained mucosal absorption barrier during epithelial shedding in asthma. This is especially so these days when a popular, reductive paradigm states that bronchial epithelial repair is defective in this disease associated with epithelial desquamation (Jin et al., 2022; Kicic et al., 2010). As discussed below, in vivo physiology‐pathophysiology at epithelial regeneration may contribute explanations regarding a maintained airway barrier at epithelial shedding.

### 6.1 Inflammatory mucosal challenges cause patchy denudation in vivo

As indicated by numerous agglomerates of shed epithelial cells (Creola bodies) in sputum, loss of epithelium is much increased and exceedingly patchy in severe exacerbating asthma (Naylor, 1962). Patchy epithelial loss in healthy airways may extend to different environmental challenges (reviewed in Persson, 2019b). However, common sectioning of bronchial tissue may overestimate the area of denudation (Ordoñez et al., 2000), especially when inflammation has produced an abnormally fragile epithelium. In accord, sectioning of inflamed guinea‐pig trachea produced >50 times greater area of denudation compared to the actual denuded area revealed by scanning electron microscopy data (Erjefält et al., 1997b). Indeed, inflammatory challenge‐induced epithelial loss left minimal craters with epithelial loss from which inflammatory exudate including neutrophils protruded (Erjefält et al., 1997a, 1997b). The patchy shape alone, aided by a locally tethered exudate and rapid epithelial regeneration at the bottom of craters (Erjefält et al., 1997b), would reduce barrier dysfunction at epithelial loss.

The craters of challenge‐induced epithelial loss appeared to be inflammatory hot spots. Hence, pathogenic significance of the mere loss of epithelium needed examination. Several further questions also arose: If there is no bleeding, what other effects are produced on the juxtaposed microcirculation? How will epithelial cells react when they lose neighboring epithelium? What is the speed of epithelial regeneration in vivo following non‐sanguineous epithelial loss? …

## 7 EPITHELIAL REGENERATION IN VIVO OCCURS IN A PLASMA EXUDATE MILIEU

**FIGURE 3:**
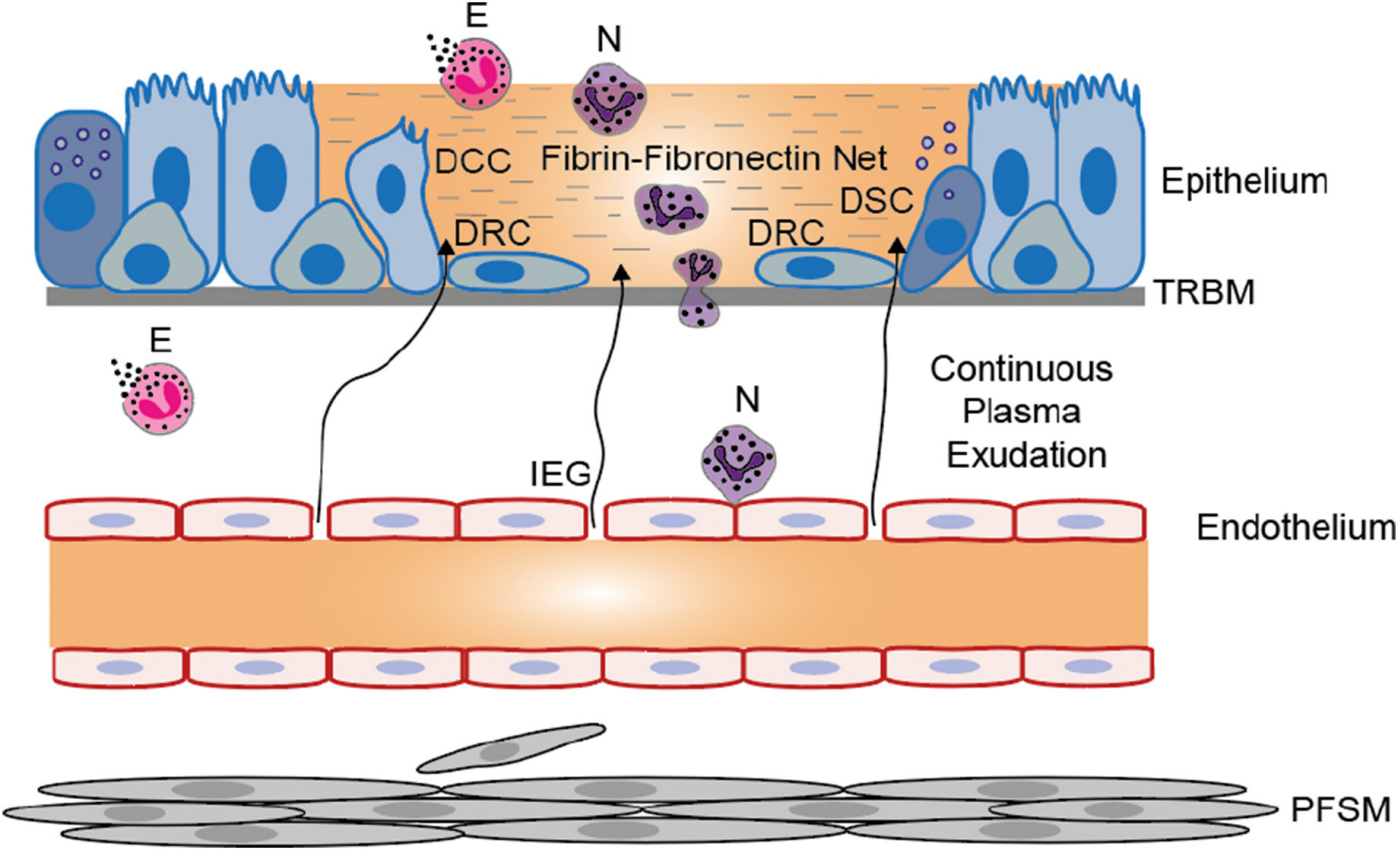
Non‐sieved plasma exudation (arrow lines) at patchy sites of epithelial loss caused by inflammatory challenges or merely by mechanical removal of epithelium, both interventions being without injury to the basement membrane and without bleeding. The denuded patches are promptly covered and by a neutrophil‐rich plasma exudate gel kept together by a fibrin‐fibronectin net. The gel‐cover is maintained by exuded plasma that provides a molecular milieu in which apparently all epithelial cell types bordering the denuded patch promptly dedifferentiate into rapidly migrating repair cells. Ciliated cells, secretory cells, and basal cells are illustrated. Epithelial repair also induces inflammatory and remodeling effects. The figure is a slight modification of a previously published version (Persson, 2019b). DCC, Dedifferentiating ciliated cell internalizing or shedding its cilia, flattening, and migrating; DRC, dedifferentiated mesenchymal‐like regeneration cells migrating speedily; DSC, dedifferentiating secretory cell releasing its secretory granules, flattening, and migrating; *E*, eosinophils undergoing primary cytolysis liberating its protein‐releasing granules; IEG, venular inter‐endothelial gaps through which non‐sieved plasma proteins extravasate; *N*, neutrophils; PFSM, proliferating fibrocytes/smooth muscle cells; TRBM, thickened reticular basement membrane.

Insight into in vivo events, induced by the mere loss of epithelial cells, was gained from studies involving non‐sanguineous, mechanical removal of epithelium leaving a well‐defined, small area of naked, intact basement membrane in guinea‐pig trachea (Erjefalt et al., 1995a). The loss of epithelial cells promptly produced venular endothelial gaps through which bulk plasma exited from the underlying microcirculation. At distinct venular sites, neutrophils extravasated and became activated. As a result, plasma proteins soon covered the denuded, intact epithelial basement membrane producing and maintaining a provisional protective barrier kept together by a neutrophil‐rich fibrin‐fibronectin net (Erjefalt et al., 1994, 1996) (Figure 3).

The humoral and cellular exudative effects, produced solely by epithelial removal, agreed with observations in inflammatory stimulus‐induced tiny craters of epithelial loss (Erjefält et al., 1997a). However, the observations may be difficult to reconcile with in vitro studies in the field. For example, there was no sign that fibronectin was produced by epithelial cells in the area of interest (Erjefalt et al., 1994). Hence, a paradigm of epithelial repair studies in vitro (Kicic et al., 2010) was not supported. In agreement with a well‐controlled duration, plasma protein exudation stops and the neutrophil‐rich plasma protein cover gel is shed as soon as a new cover of undifferentiated repair cells is established (Erjefalt et al., 1994).

## 8 SPEEDY EPITHELIAL REGENERATION IN VIVO

A range of additional, prompt responses were induced by the controlled, non‐sanguineous denudation. Epithelial cells bordering the denuded area dedifferentiated into flattened repair cells that, tethered, migrated at a speed of 2–3 μm/min across the intact basement membrane. As originally demonstrated in vivo (Erjefalt et al., 1995a), repair stem cells thus emanated not only from basal cells (Ruysseveldt et al., 2021) but also from different types of the human‐like pseudostratified epithelium including ciliated and secretory cells (Figure 3). It is suggested that this mode of recruiting stem cells would be an optimal way of repairing a broken lining of airways pseudostratified epithelium. The high speed of epithelial cell regeneration agrees with the observation that the basement membrane at the bottom of inflammation‐induced mini‐craters of epithelial loss are almost fully covered by repair cells within an hour (Erjefält et al., 1997b). The in vivo morphology observations (Erjefalt et al., 1995a), revealing stem cell functions of the common types of epithelial cells that surround the denuded patches, are supported by recent molecular definition of cell types and their plasticity (Montoro et al., 2020; Sikkema et al., 2023).

## 9 POSSIBLE RECONCILIATION OF HUMAN IN VIVO DATA ON EPITHELIAL INTEGRITY

Given the anatomical and physiological similarities between guinea‐pig tracheal and human airway mucosa, it is suggested that this concordance may also extend to epithelial regeneration. Consistent with this possibility, a plasma‐derived physical and biological barrier, promoting rapid epithelial regeneration at small patches of denudation tallies with the lack of overt barrier defect in vivo in a desquamatory disease like asthma. Furthermore, the prompt restitution of epithelium in vivo would reconcile early enigmatic in vivo observations in the infected nose of humans where epithelial shedding was induced (Turner et al., 1982), but examination of mucosal histology failed to detect any breach of epithelial linings (Winther et al., 1984). Similarly, loss of bronchial epithelial cells may occur in asthma (Naylor, 1962) although careful analyses of bronchial biopsies may not show denuded areas (Ordoñez et al., 2000).

## 10 EPITHELIAL REGENERATION COMES AT PATHOPHYSIOLOGICAL COST

If effects on the epithelial barrier are as limited as discussed above it seems reasonable to ask whether other effects, induced solely by loss of epithelium, can be pathogenic? Beside evoking plasma protein exudation, it emerged that epithelial loss‐regeneration alone, in otherwise healthy airways, induces eosinophil activation (by primary lysis as in asthma; Persson & Uller, 2013) and neutrophil recruitment and activation in the affected area (Erjefalt et al., 1996). Furthermore, proliferation of fibroblast and airway smooth muscle cells, mucus hypersecretion and remodeling of epithelium and its basement membrane are induced (Persson & Erjefält, 1997). The composite mucosal responses (Figure 3) actually overlap with several parts of the pathophysiology and pathology of bronchi that have been observed in patients with asthma. The latter include remodeling of the epithelial basement membrane and proliferation of smooth muscle (Figure 3). If numerous patches of epithelial loss are induced, as demonstrated by Naylor (1962), microvascular and other responses to epithelial loss‐regeneration could become of pathogenic significance potentially contributing to the inception of asthma and the development of its severity (Al Heialy et al., 2022; Persson, 2022a, 2022b). It has recently been more widely realized that roles of the plasma exudation responses in airways with intact and repairing epithelia is both overlooked and under‐researched (Al Heialy et al., 2022).

## 11 CONCLUSION

It is widely understood that translation of preclinical research to human in vivo conditions is fraught with uncertainties. Although less accepted as a research strategy, the reverse also applies, that is, exploratory studies involving humans in vivo is a source of unexpected discoveries often challenging current concepts (Persson, 1999, 2019a; Persson et al., 2001). Original in vivo findings can thus be rather more consequential, and relevant, than the general utility of checking the applicability of in vitro‐based notions to the more complex situation in vivo. The present article review falls within the former category. Physiological interaction between a superficial microcirculation and a pseudostratified epithelial lining evidently moves unfiltered blood plasma solutes to the surface of intact airways. The transmission is a prompt response to inflammatory mucosal challenges. Without size restriction and without reducing the epithelial barrier that protects against penetrability of inhaled toxins, the armamentarium of circulating peptide/proteins (with established defense and repair actions; not reviewed here) appear on the airway surface at sites of challenge. The plasma exudation responses are well‐controlled with regard to reversibility, repeatability, magnitude, and duration. These features, applying to human airways, provide a framework for interpretation of plasma peptides/proteins in clinical samples of airway surface liquids. A role of plasma exudation as a first line antimicrobial defense emerges. If provocations involve loss of epithelial cells from an intact basement membrane, there is separate, local induction of plasma exudation, which is well‐controlled in terms of localization, magnitude, and duration. This response, demonstrated in human‐like airway mucosa, creates a provisional barrier, and molecular milieu, protecting and promoting optimal repair in vivo during the most vulnerable phase of epithelial regeneration. Since current concepts regarding antimicrobial respiratory defense and epithelial regeneration mechanisms often are based on models where plasma exudation is absent, the present in vivo perspective is suggested as a basis for future multifaceted research in these topical fields.

## DEDICATION

Honoring the memory of the late Morgan Andersson and Ingrid Erjefält, clinical and experimental researchers in Lund – we worked together in this field over decades.
